# Pulsed Electric Field-Assisted Extraction of Aroma and Bioactive Compounds From Aromatic Plants and Food By-Products

**DOI:** 10.3389/fnut.2021.792203

**Published:** 2022-01-27

**Authors:** Serena Carpentieri, Anet Režek Jambrak, Giovanna Ferrari, Gianpiero Pataro

**Affiliations:** ^1^Department of Industrial Engineering, University of Salerno, Fisciano, Italy; ^2^Faculty of Food Technology and Biotechnology, University of Zagreb, Zagreb, Croatia; ^3^ProdAl Scarl, University of Salerno, Fisciano, Italy

**Keywords:** pulsed electric fields (PEF), extraction, green solvents, bioactive compounds, aroma compounds

## Abstract

In this work, the effect of pulsed electric field (PEF) pre-treatment on the extractability in green solvents (i. e., ethanol–water mixture and propylene glycol) of target aroma and bioactive compounds, such as vanillin from vanilla pods, theobromine and caffeine from cocoa bean shells, linalool from vermouth mixture, and limonene from orange peels, was investigated. The effectiveness of PEF as a cell disintegration technique in a wide range of field strength (1–5 kV/cm) and energy input (1–40 kJ/kg) was confirmed using impedance measurements, and results were used to define the optimal PEF conditions for the pre-treatment of each plant tissue before the subsequent solid–liquid extraction process. The extracted compounds from untreated and PEF-treated samples were analyzed *via* GC-MS and HPLC-PDA analysis. Results revealed that the maximum cell disintegration index was detected for cocoa bean shells and vanilla pods (*Z*_p_ = 0.82), followed by vermouth mixture (*Z*_p_ = 0.77), and orange peels (*Z*_p_ = 0.55). As a result, PEF pre-treatment significantly enhanced the extraction yield of the target compounds in both solvents, but especially in ethanolic extracts of vanillin (+14%), theobromine (+25%), caffeine (+34%), linalool (+114%), and limonene (+33%), as compared with untreated samples. Moreover, GC-MS and HPLC-PDA analyses revealed no evidence of degradation of individual compounds due to PEF application. The results obtained in this work suggest that the application of PEF treatment before solid–liquid extraction with green solvents could represent a sustainable approach for the recovery of clean labels and natural compounds from aromatic plants and food by-products.

## Introduction

Nowadays, the food, pharmaceutical, and cosmetic industries are continuously striving to meet the evolving consumer demands for clean labels and natural compounds with functional and health beneficial properties (flavorings, colorants, antioxidants) (1, 2). In this line, the plant extracts market is increasingly catching on, and the preferences for natural products are expected to drive its growth during the coming years (3).

For example, vanilla pod is one of the most widely used flavoring agent in food products, beverages, cosmetics, and pharmaceutical preparations, with vanillin, a phenolic aromatic aldehyde, being its major flavor constituent. Apart from its aroma, vanillin and vanilla extracts have been reported to have beneficial properties, such as antioxidant, anti-mutagenic, hypolipidemic, and anticarcinogen activity (4). Another widespread valuable flavor compound is linalool, which represents approximately 70% of the terpenoids of a wide variety of herbal scents (5). In particular, linalool is among the most abundant compounds present in the herbal mixture at the basis of vermouth wine production (6). Several studies have been published reporting different biological activities of linalool, such as central nervous system depressant effects, as well as analgesic and anti-inflammatory activities (5, 7).

In addition to aromatic plants, several food by-products also represent a cheap and rich source of valuable intracellular compounds, that if properly recovered, could have great potential in industrial applications as food supplements or nutraceutical ingredients. In this framework, the citrus industry is a major contributor to the food by-products, with juice processing plants generating a large amount of peels, which account for up to 60% of the total fruit weight (8). However, orange peel also contains a wide variety of valuable compounds, dietary fibers, polyphenols, and essential oils, thus providing the opportunity for its valorization as a cost-effective source of high value-added compounds (8, 9). Among them, limonene, a lipophilic monoterpene, is one of the main constituents of orange peel (up to 4% on a dry basis). Due to its antioxidant properties and aroma, limonene plays a key role in the global market for industrial applications (8).

An additional interesting source of bioactive compounds of industrial growing interest is represented by residues of cocoa beans, which are especially rich in alkaloids, mainly theobromine and caffeine (10, 11). These compounds are of nutraceutical and pharmacological interest due to their stimulatory effects on the nervous, gastrointestinal, vascular, and respiratory systems (10).

The extraction and recovery of aroma and bioactive compounds from these plant matrices is typically performed *via* conventional solvent extraction techniques, often using hazardous petrochemical solvents, or *via* their synthetic production. As an example, 85% of the global supply of vanillin comes from petroleum-derived compounds, being more cost and time effective compared to the traditional solvent extraction processes (12). On the other hand, conventional extraction methods are typically time and energy consuming, and require excessive usage of organic solvents, which are mostly toxic and harmful (13). In light of these drawbacks, recently more sustainable, efficient, rapid, and environmentally friendly extraction techniques based on the use of green solvents coupled with emerging technologies, such as microwave, ultrasound, and pulsed electric field (PEF), were proposed to reduce the mass transfer resistances of target solutes and solvents through the cell envelop (membrane, wall) (1).

In particular, it is known that the application of PEF pre-treatment to plant tissues, which consists in exposing plant material placed between two metal electrodes to repetitive short-duration pulses (1 μs−1 ms) of moderate electric field (0.5–10 kV/cm) and relatively low energy input (1–20 kJ/kg), induces the permeabilization of cell membranes by pores formation, known as electroporation or electropermeabilization (14). This has shown a great potential to intensify the selective recovery of target intracellular compounds (15), while reducing the energy costs, the solvent consumption, and shortening the treatment time (1, 16).

However, to date, only few works demonstrated the feasibility of PEF technology to improve the recovery yield of phenolic compounds and caffeine from cocoa bean shells (10), phenolic compounds from orange peels (9, 17, 18), and aromatic plant matrices (19–22), but none of them were addressed to the extractability of limonene from orange peels, as well as aroma and bioactive compounds from vanilla pods and vermouth mixture.

Furthermore, it should be emphasized that any comparison of the extraction efficiency of valuable compounds from different plant matrices is very difficult being currently based on literature data achieved using different types of equipment and experimental protocols. Therefore, because of a full exploitation of PEF technology at the industrial level, there is a need to obtain comparable experimental data, which will allow to identify the most suitable plant materials for PEF treatment as well as to set processing guidelines.

The main aim of this study was to investigate the potential of PEF pre-treatment in combination with solid–liquid extraction (SLE) to intensify the extractability of target aroma and bioactive compounds, such as vanillin from vanilla pods, linalool from vermouth mixture, theobromine and caffeine from cocoa bean shells, and limonene from orange peels, using the same equipment and experimental protocols. Specifically, the effect of different combinations of electric field strength (E) and total specific energy input (W_T_) on the cell disintegration index of these plant tissues was evaluated to define the optimal PEF pre-treatment conditions to be applied before the subsequent SLE process. The latter was conducted by using solvents with low environmental impact and toxicity, like ethanol–water mixtures and propylene glycol (23, 24). Then, the effect of PEF on the recovery of target compounds in the extracts was evaluated by performing HPLC-PDA and GC-MS analyses.

## Materials and Methods

### Chemicals and Raw Materials

Ethanol, propylene glycol, and all reagents and standards involved in HPLC-PDA, and GC-MS analyses were purchased from Sigma–Aldrich (Steinheim, Germany).

Plant materials, such as vanilla pods, cocoa bean shells, vermouth mixture, and orange peels, with a moisture content on a wet basis of 9.9 ± 0.7, 8.9 ± 0.8, 7.7 ± 1.0, and 8.7 ± 1.1%, respectively, were provided by Kerry Ingredients & Flavours Italia S.P.A (Mozzo, Bergamo, Italy). The samples were stored in polyethylene bags kept under vacuum, in a dark and dry place until use.

Before each PEF experiment, dry samples were subjected to re-hydration by immersion in distilled water. The suspension with a solid to liquid ratio of 1:50 (g/ml), was kept under gentle magnetic agitation (140 rpm) at 25°C, for up to 30 min, which was a long enough time to ensure the full rehydration of the plant matrices. The final moisture content of the rehydrated plant materials on a wet basis was 70.8 ± 2.1% for vanilla pods, 69.6 ± 1.5% for cocoa beans, 73.5 ± 0.9% for vermouth mixture, and 67.6 ± 1.1% for orange peels.

### PEF Apparatus

Pulsed electric field treatments of each rehydrated plant matrix, before either impedance analysis or solid–liquid extraction (SLE) process, were performed using a laboratory-scale batch system previously described elsewhere (25). Briefly, the system consisted of a high voltage pulsed power (25 kV−500 A) generator (Modulator PG, ScandiNova, Uppsala, Sweden) able to deliver monopolar square wave pulses with a different pulse width (3–25 μs) and frequency (1–450 Hz). The generator was electrically connected to a batch treatment chamber, made of two plane-parallel electrodes of stainless steel separated by a Teflon spacer. The distance between the two electrodes was 2 cm, and their area was 75 cm^2^. The actual voltage and current signals at the treatment chamber were measured, respectively, by a high voltage probe (Tektronix, P6015A, Wilsonville, OR, United States) and a Rogowsky coil (2-0.1, Stangenes, Inc., United States), connected to an oscilloscope (Tektronix, TDS 3034B, Wilsonville, OR, United States). The maximum electric field intensity (E, in kV/cm) was evaluated as the peak voltage divided by the inter-electrode gap. The total specific energy input (W_T_, in kJ/kg of rehydrated plant tissues) was calculated according to Equation (1) (22):


(1) $$W_{T}=\frac{n}{m_{RE}}\int_{0}^{\infty }U\left(t\right)\;\cdot \;I\left(t\right)dt$$

where *U(t)* and *I(t)* represent the voltage across the electrodes and the current intensity through the treated product at time *t*, respectively, *n* is the number of pulses applied, and *m*_*RE*_ is the mass of the treated rehydrated plant material.

### Quantification of PEF-Induced Cell Membrane Permeabilization

The cell disintegration index (*Z*_p_) was determined to quantify the degree of cell membrane permeabilization of each plant tissue induced by PEF treatment before SLE process. This index has been successfully used as a reliable macroscopic indicator of the degree of cell membrane permeabilization in diverse plant tissues and to select the optimal PEF treatment conditions (13, 22, 26–30).

In this work, the determination of *Z*_p_ *via* impedance analyses was carried out according to the method described by Bobinaite et al. (28), with some modifications. For each plant material, measurements of the electrical complex impedance of untreated and PEF-treated samples were carried out by loading about 5 g of the rehydrated sample into a measuring cell, which consisted of two parallel plate cylindrical electrodes (3 cm in diameter) separated by a polycarbonate tube (1 cm electrode gap). The electrodes were connected to an impedance analyzer (Solartron 1260, United Kingdom), which was working in the frequency range of 10^2^-10^7^ Hz. PEF treatments were carried out at different field strengths (E = 1, 3, and 5 kV/cm) and total specific energy input (W_T_ = 1, 5, 10, 15, 20, and 40 kJ/kg), at a constant pulse repetition frequency (5 Hz) and pulse width (20 μs). The initial temperature of the samples was set at 20 ± 1°C and no appreciable temperature increase was detected due to the relatively low energy input delivered during the treatment.

For each PEF treatment condition investigated, the *Z*_*p*_ value was calculated on the basis of the measurement of the absolute value of the complex impedance |**Z**| of untreated (|**Z**_**untr**_|) and electrically treated tissue (|**Z**_**tr**_|), in the low (0.1 kHz) and high (1 MHz) frequency ranges, using Equation (2) (31).


(2) $$Z_{p}=\;\frac{\left|Z_{untr\;(o.1\;kHz)}\right|-\left|Z_{tr\;(0.1\;kHz)}\right|}{\left|Z_{untr\;(0.1\;kHz)}\right|-\left|Z_{tr\;(10\;MHz)}\right|}$$

The *Z*_*p*_ value varies between 0 (for intact tissues) and 1 (for fully permeabilized tissue). All the measurements were carried out in triplicate.

The achieved *Z*_p_ values were also used to define optimal treatment conditions in terms of field strength (*E*_*opt*_) and energy input (*W*_*T, opt*_), which enabled the achievement of the highest degree of cell membrane permeabilization with the minimum treatment severity. These optimal conditions were applied during the subsequent PEF-assisted SLE experiments.

### PEF-Assisted Extraction Experiments

For PEF-assisted extraction experiments, ~50 g (on average) of each rehydrated plant material was loaded into the treatment chamber and PEF pre-treated under the optimal conditions (*E*_*opt*_*, W*_*T, opt*_) previously defined through the Z_p_ determinations. After the electro-permeabilization treatment, the samples were subjected to SLE process in two different solvents, namely, ethanol–water mixture and propylene glycol. Preliminary tests enabled to define optimal extraction conditions (data not shown), as summarized in Table 1, in terms of type and concentration of solvent, solid–liquid ratio, temperature, and extraction time, which were sufficient to achieve significant extraction yields of the target intracellular compounds. Specifically, during the SLE process, the PEF-treated samples were immediately placed into a glass flask, added with a given amount of the extracting solvent (Table 1), and then introduced in an orbital incubator S150 (PBI International, Milan, Italy), where the extraction process was carried out under shaking at 160 rpm.

**Table 1:** Operative conditions used for the solvent extraction of aromas and bioactive compounds from the selected plant matrices.

**Matrix**		**Target compound**	**Extraction solvent**	**Extraction conditions**
Vanilla pods	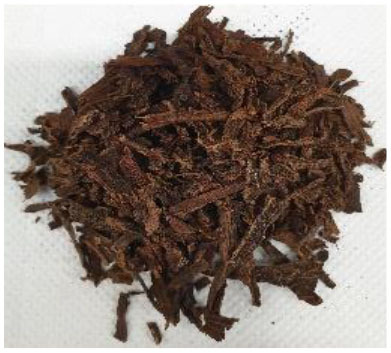	Vanillin	60% Ethanol-water (v/v)	S/L = 1:20 (g/ml)
				T = 25°C
				Time = 3 h
			Propylene glycol	S/L = 1:20 (g/ml)
				T = 25°C
				Time = 3 h
Cocoa bean shells	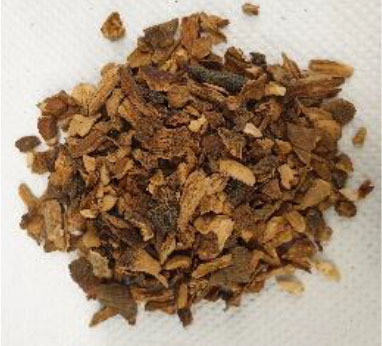	Caffeine	40% Ethanol-water (v/v)	S/L = 1:30 (g/ml)
				T = 40°C
				Time = 2 h
		Theobromine		
			Propylene glycol	S/L = 1:30 (g/ml)
				T = 40°C
				Time = 2 h
Vermouth mixture	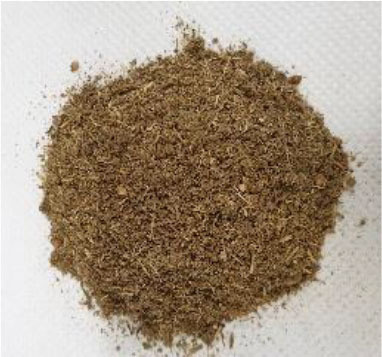	Linalool	99.9% Ethanol-water (v/v)	S/L = 1:20 (g/ml)
				T = 40°C
				Time = 4 h
			Propylene glycol	S/L = 1:20 (g/ml)
				T = 40°C
				Time = 4 h
Orange peels	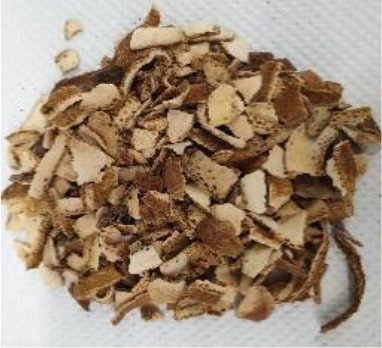	Limonene	99.9% Ethanol-water (v/v)	S/L = 1:20 (g/ml)
				T = 40°C
				Time = 3 h
			Propylene glycol	S/L = 1:30 (g/ml)
				T = 40°C
				Time = 3 h

*S/L, solid to liquid ratio*.

For the sake of comparison, the conventional SLE process was carried out using the same protocol without PEF treatment.

At the end of the diffusion step, the extracts from untreated and PEF-treated samples were centrifuged at 5,289 × g (PK130R model, ALC International, Cologno Monzese, Italy) for 10 min at 4°C to separate the supernatants. The latter were then filtered with 0.45 μm syringe filters and subsequently stored at −20°C until further analysis.

### Analysis of the Extracts

#### Quantification of Linalool and Limonene by GC-MS

The identification and the amount of limonene and linalool contained in orange peels and vermouth extracts, respectively, was carried out by Gas Chromatography coupled with Mass Spectroscopy (GC-MS) analysis, using the method described by Radünz et al. (32) with some modifications.

A 10 ml of vermouth mixture extract was further extracted three times with ether/pentane solution (50%, v/v), centrifuged at 1,000 rpm for 10 min, filtered, anhydrified with sodium sulfate, and then 5 μl injected in the GC-MS system [GC-MS Thermo system equipped with a Restek Trx-5Sil MS column (30 m × 0.25 mm × 0.25 μm); Restek Corporation, United States].

For limonene quantification, 0.5 ml of orange peels extract was mixed with 20 μl of hexane and 1 ml of acetonitrile, and centrifuged at 1,000 rpm for 10 min. The supernatant was collected, mixed to 0.5 ml of hexane, and centrifuged at 1,000 rpm for 10 min. Then, 5 μl of the obtained upper hexane layer was injected into the column.

For either linalool or limonene analysis, 10 μl of helium was used as the carrier gas at a flow rate of 1.5 ml/min. The injector was operated in splitless mode at 250°C and the ion source at 250°C. The oven temperature was initially maintained at 70°C for 10 min, then gradually increased to 150°C, and then kept constant for 5 min. The mass spectrometer was operated in electron ionization mode with an electron energy of −70.1 eV (electron volt). The mass scan range employed was 50–650 amu (single atomic mass unit). Limonene and linalool were identified by the NIST official library as well as by comparing their GC-MS retention times with those of commercial standards and their concentration was expressed as mg/100g of dry weight (DW) of plant sample. All the standards were dissolved in the diluent solution (ranging from 1 to 10 mg/L) to generate five-point standard calibration curves (R^2^ = 0.999).

#### Quantification of Vanillin, Caffeine, and Theobromine by HPLC-PDA

The High-Performance Liquid Chromatography-Photodiode Array Detection (HPLC-PDA) analyses of vanillin (from vanilla pods extracts) and caffeine and theobromine (from cocoa bean shells extracts) was performed using a Waters 1525 Separation Module equipped with a photodiode array detector Water 2996 (Waters Corporation, United States), following the methodology described by Frontuto et al. (13), with some modifications.

Analytical separation of vanillin, caffeine, and theobromine was carried out in a Waters Spherisorb C18 reverse-phase column (5 μm ODS2, 4.6 × 250 mm, Water Corporation, United States). Before the HPLC-PDA analysis, the extracts were filtered with 0.20 μm filters and then diluted with an ethanol–water solution (50%, v/v). The mobile phase consisted of (A) acetic acid in water (0.1 %, v/v), and (B) acetonitrile/acetic acid (99.9:0.1, v/v). For compounds separation, the following gradient was used: 0–10 min 95% eluent A (5% eluent B), 10–13 min 100% eluent B, 13–17 min 95% eluent A (5% eluent B). The injection volume and the flow rate of the mobile phase were 10 μl and 0.6 ml/min, respectively. The signal for the quantification of vanillin, caffeine, and theobromine was recorded at 280, 273, and 272 nm, respectively. All commercial standards were dissolved each into the respective extraction solvent to generate 6-point standard calibration curves (R^2^ = 0.999). The concentration range was from 5 to 200 mg/L for caffeine and theobromine, and from 5 to 300 mg/L for vanillin. The results were expressed as milligrams of target compounds (vanillin, caffeine, or theobromine)/100 g of dry weight (DW) of plant sample.

### Statistical Analysis

All the experiments and analyses of the obtained extracts were performed in triplicate and the results were reported as means ± standard deviations. Differences among mean values were analyzed by one-way ANOVA, by using SPSS 20 (SPSS IBM., Chicago, IL, United States) statistical package. Tukey's test was performed to determine statistically significant differences (*p* < 0.05).

## Results and Discussion

### Effect of PEF Treatment on the Cell Membrane Permeabilization of Plant Tissues

Data reported in Figure S1 revealed that the extent of cell membrane permeabilization increased with increasing the PEF treatment severity. In particular, according to previous findings (13, 16, 17, 22, 28–30, 33), regardless of the field strength applied, *Z*_*p*_ value increased with increasing the energy input up to a saturation point dependent on the type of plant matrices, above which no further cellular damages could be detected. Moreover, at any energy level, the increase in the electric field strength from 1 kV/cm up to 3 kV/cm significantly (*p* < 0.05) increased the *Z*_*p*_ value, while the application of PEF treatment at the highest field strength (5 kV/cm) produced an additional increase (*p* < 0.05) in the extent of cellular damages, but only for orange peel tissue.

From these results, optimal PEF treatment conditions, with regard to the minimal electric field strength (E_opt_, kV/cm) and total specific energy input (W_T, opt_, kJ/kg) that enabled the achievement of the highest degree of cell membrane permeabilization, were defined for each investigated plant matrix, as summarized in Table 2. In particular, the maximum *Z*_*p*_ value (0.82) was detected for the PEF-treated samples of vanilla pods and cocoa beans residues at 3 kV/cm and 20 kJ/kg. Vermouth mixture, instead, required slightly lower values of energy input (15 kJ/kg) at the same field strength (3 kV/cm) to augment noticeable cell membrane permeabilization degree up to similar *Z*_*p*_ levels (0.77). On the other hand, the highest resistance to electropermeabilization treatment was shown by orange peels, which exhibited the lowest *Z*_*p*_ value (0.55) despite the application of a PEF treatment at 5 kV/cm and up to 40 kJ/kg. This might be ascribed to the highly fibrous nature of orange peels (largely composed of cellulose, hemicellulose, pectin, lignin) and their apparent rubbery texture due to the presence of pectin as the predominant polysaccharide in the cell wall (34), which might hinder the permeabilization of the cell envelop.

**Table 2:** Optimal PEF treatment conditions in terms of electric field strength (E, in kV/cm) and total specific energy input (W_T_, in kJ/kg), enabling the highest cell disintegration index (*Z*_*p*_) for all the investigated matrices.

**Treated matrix**	**Optimal PEF conditions**		***Z_***p***_***
	**E_**opt**_ (kV/cm)**	**W_**T, opt**_ (kJ/kg)**	
Vanilla pods	3	20	0.82 ± 0.07
Cocoa bean shells	3	20	0.82 ± 0.05
Vermouth mixture	3	15	0.77 ± 0.10
Orange peels	5	40	0.55 ± 0.09

*PEF, pulsed electric field*.

As per the literature survey, no previous work focused on the influence of PEF treatment on the cell disintegration degree of cocoa bean shells, vanilla pods, and vermouth mixture tissues, while only one work investigated the permeabilization effect of PEF on orange peel tissues. This makes even more difficult any comparison with data found in the current literature on different plant materials, also due to the different types of equipment and experimental conditions used. Nevertheless, the general trend of the influence of electric field strength and total specific energy input on *Z*_*p*_ values observed in this research is somehow consistent with previously reported data for other plant tissues, such as blueberries, spearmint, oregano, thyme, orange peels, and tomato peels (13, 17, 22, 29, 30). As an example, Luengo et al. (17) found the highest value of *Z*_*p*_ of 0.33 for the most intense treatment conditions tested (7 kV/cm, 113 kJ/kg), concluding that the permeabilization of orange tissue requires applying more intense PEF treatments than for other vegetable tissues.

Therefore, the results obtained from this investigation, using the same equipment and experimental protocol, demonstrated that the PEF treatment was able to efficiently induce the cell membrane permeabilization of different plant tissues, but to an extent dependent on the type of matrices.

According to these results, further investigations of PEF pre-treatment on the extractability of aroma and bioactive compounds from cocoa beans residues, vanilla pods, vermouth mixture, and orange peels were carried out under the optimal conditions reported in Table 2.

### Effect of the PEF Pre-treatment on the Extraction of Aromas and Bioactive Compounds

#### Content and Composition of Vermouth and Orange Peels Extract

**Figure 1:**
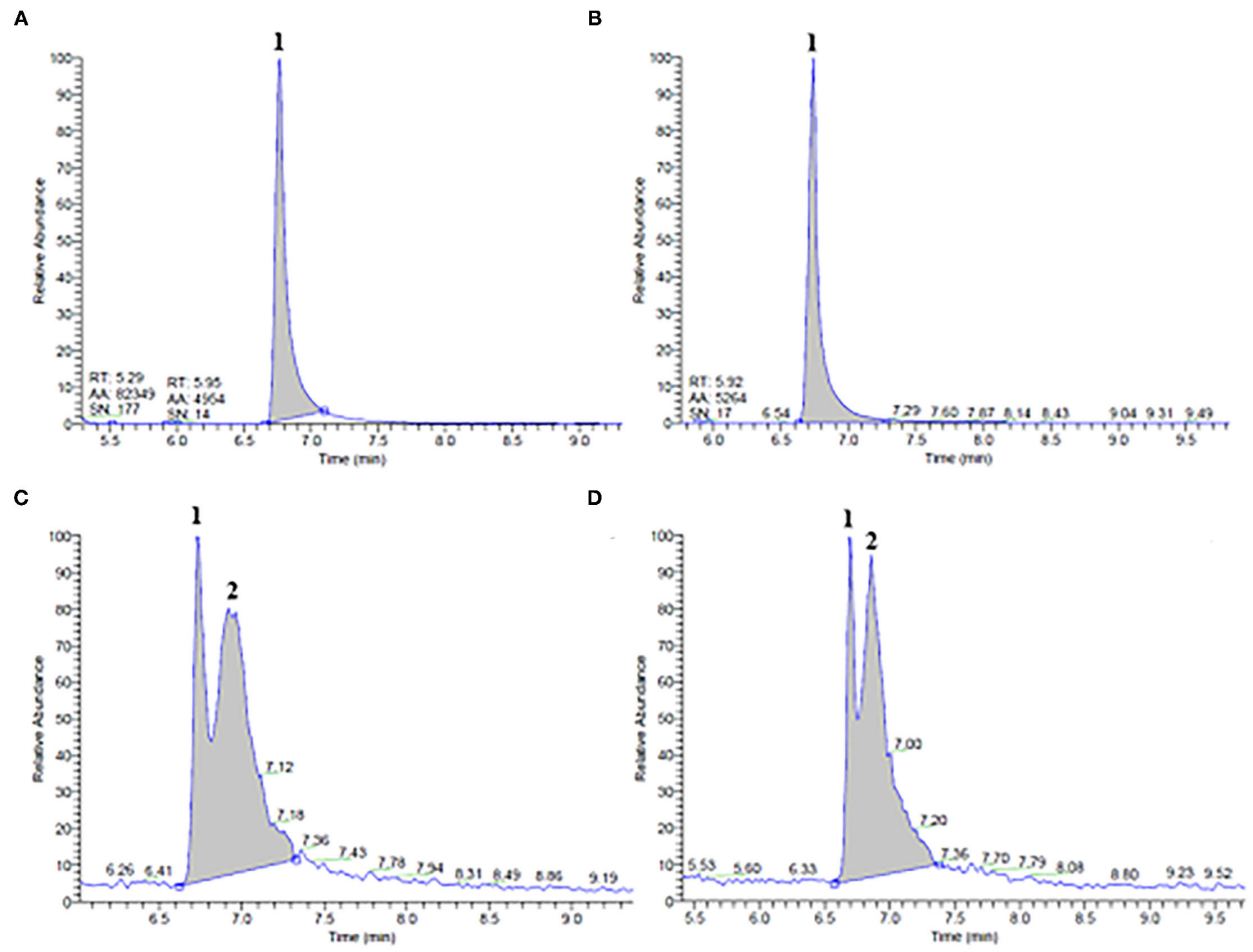
GC/MS chromatograms of ethanol **(A,B)** and propylene glycol **(C,D)** extracts obtained from **(A,C)** untreated (control) and **(B,D)** pulsed electric field (PEF)-treated (E_opt_ =5 kV/cm; W_T, opt_ = 40 kJ/kg) orange peels. Peak identification: Limonene (1); unidentified compound (2).

**Figure 2:**
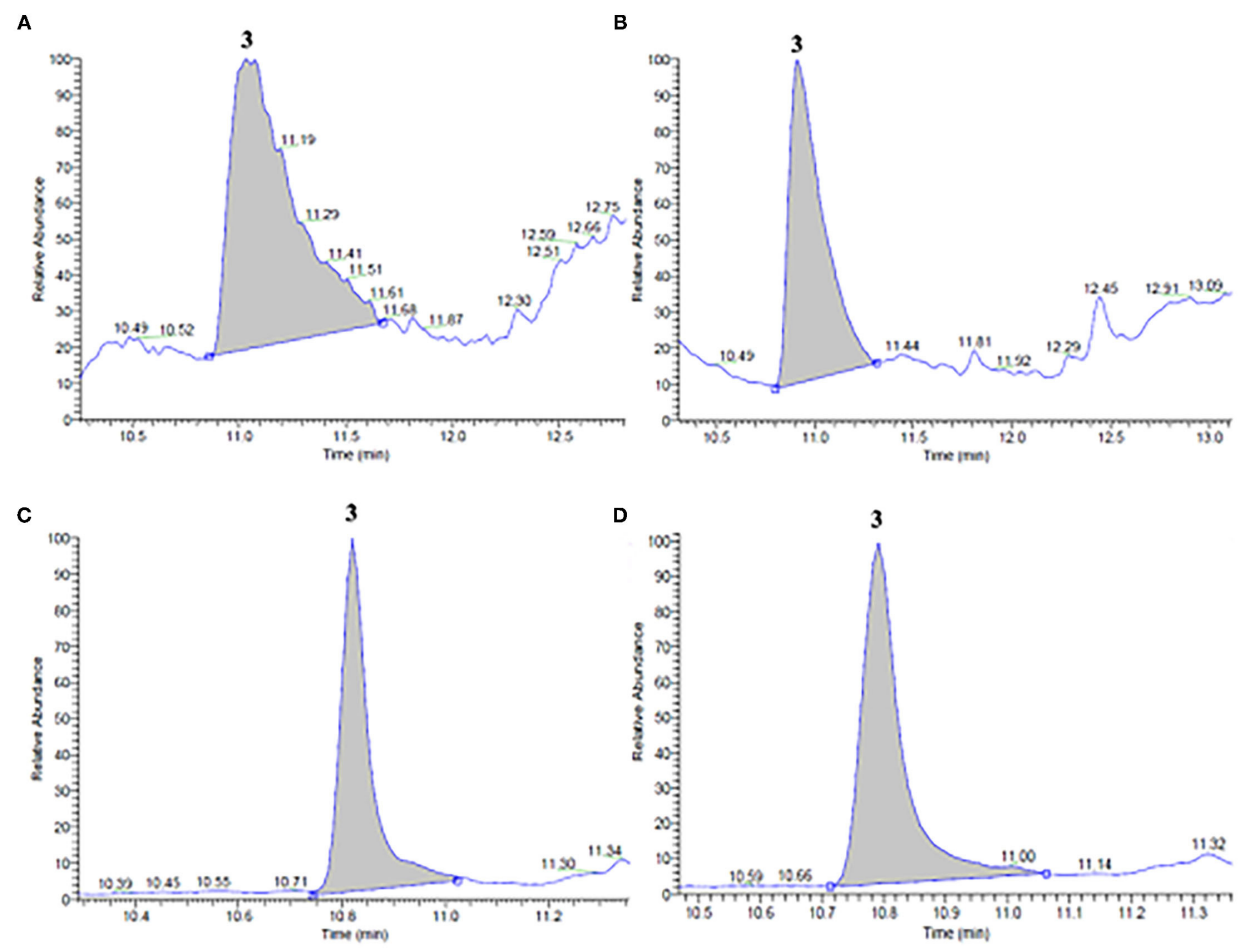
GC/MS chromatograms of ethanol **(A,B)** and propylene glycol **(C,D)** extracts obtained from **(A,C)** untreated (control) and **(B,D)** PEF-treated (E_opt_ =3 kV/cm; W_T, opt_ = 15 kJ/kg) vermouth mixture. Peak identification: Linalool (3).

Using GC-MS measurements, limonene and linalool were identified and quantified in both ethanol and propylene glycol extracts of untreated and PEF-treated orange peels and vermouth mixture, respectively, as reported in Figures 1, 2, and Table 3.

**Table 3:** Concentrations (in mg/100g_DW_) of limonene and linalool detected *via* GC-MS analysis in the ethanol and propylene glycol extracts from the untreated and PEF-treated orange peels and vermouth mixture, respectively.

**Peak no**.	**Compound**	**Retention time (min)**	**Extraction solvent**	**Concentration (mg/100g**_**W**_ **)**	
				**Untreated**	**PEF treated**
1	Limonene	6.76	Ethanol	3.0 ± 0.2^aB^	4.0 ± 0.1^bB^
		6.71	Propylene glycol	0.6 ± 0.1^aA^	0.8 ± 0.3^aA^
3	Linalool	10.97	Ethanol	1.4 ± 0.3^aA^	3.0 ± 0.2^bB^
		10.80	Propylene glycol	1.5 ± 0.4^aA^	1.7 ± 0.3^aA^

*PEF, pulsed electric field*.

*Data are expressed as means ± standard deviation*.

*PEF treatments carried out under optimal condition are reported in Table 2*.

*Different lowercase letters in the same line indicate significant differences between the samples (p ≤ 0.05). Different uppercase letters within the same column indicate significant differences between the samples (p <0.05)*.

Results of Figure 1 show that both ethanolic and glycolic extracts obtained from untreated and PEF-treated orange peels presented similar chromatogram profiles, confirming that neither selective extraction nor degradation occurred due to the application of a PEF pre-treatment. However, it is worth noting that, while only one major peak corresponding to limonene (peak 1) was detected at an elution time of 6.76 min in ethanol extracts (Figure 1A), one slightly minor and unidentified compound (peak 2), was also detected immediately after the elution time (6.71 min) of limonene (peak 1) in the glycolic extracts (Figure 1C). Moreover, as reported in Table 3, the concentration of limonene detected in ethanol and propylene glycol extracts of untreated samples was 3.0 ± 0.2 mg/100g_DW_ and 0.6 ± 0.1 mg/100_DW_, respectively. These results highlight the higher affinity and selectivity of ethanol in extracting limonene, a lipophilic cyclic monoterpene (8), as compared with propylene glycol.

Pulsed electric field pre-treatment of orange peels remarkably enhanced the limonene content in the ethanolic extracts (by 33 %) as compared with the control extraction, while a slight but not significant (*p* > 0.05) increase was detected in the case of glycolic extracts (Table 3). Moreover, it is worth noting that, in comparison with the control sample, PEF pre-treatment increased the peak area of the unidentified compound (peak 2) in glycolic extracts (Figures 1C,D). In this case, in addition to the electroporation effect induced by PEF pre-treatment, the different penetration and solubilizing capacity of the two solvents used may have played an important role in the selectivity and extractability of target intracellular compounds. Moreover, it is likely that, the well-known capability of ethanol to affect the phospholipid bilayer of biological membranes (35), may have compensated for the relatively lower permeabilization effects of PEF detected on the cells of orange peel tissues (*Z*_*p*_= 0.55), as compared with that detected for the other investigated matrices, thus leading to a significant recovery of limonene in the ethanolic extracts.

Moreover, from the chromatogram profiles of untreated vermouth mixture extracts presented in Figures 2A,C, it can be seen that, independent of the type of solvent, only one major peak corresponding to linalool (peak 3) was detected at an elution time of 10.97 min in ethanol and 10.80 min in propylene glycol extracts. These results are coherent with the fact that linalool is present in significant amounts in many plants of Lamiaceae, Lauraceae, and Rutaceae families, which make up the vermouth mixture (5). However, it is worth noting that no significant (*p* < 0.05) differences could be detected between the concentrations of linalool in ethanolic and glycolic control extracts (Table 3).

Interestingly, the application of a PEF pre-treatment before SLE process kept the original GC-MS profiles detected in the control extracts, thus demonstrating that the electrical pre-treatment induced neither selective extraction nor degradation of specific intracellular compounds. This is in agreement with the observations reported by other authors (17, 22, 30, 36–39), who found that PEF treatment did not significantly alter the chromatogram profiles of different plant tissue extracts, probably due to the relatively mild intensity of the applied treatment. As also reported in Table 3, the only differences observed were that PEF pre-treatment remarkably enhanced the linalool content, especially in the ethanolic extracts (by 114%) and at a lower extent in the glycolic extracts (13 %).

These results could be explained by the electroporation effect induced by PEF pre-treatment and the different solubilizing capacities of the two solvents involved in the extraction process. According to the data reported in Table 2, PEF pre-treatment can induce the permeabilization of cell membrane of the vermouth tissues (*Z*_*p*_= 0.77), which likely facilitated the penetration of the solvent into the cytoplasm of the plant cell and the subsequent diffusion of the solubilized compounds, thus intensifying the extractability of linalool. On the other hand, the slightly lower polarity of ethanol in comparison with propylene glycol (40), likely makes ethanol a more adequate solvent to solubilize non-polar compounds such as linalool. This is also corroborated by a previous theoretical study, in which it was shown that linalool has a good probability of solubility (52.5%) in ethanol already at room temperature (24).

Additionally, it is likely that the capability of ethanol to affect the barrier properties of the cell envelop of the plant tissues by acting on the phospholipid bilayer of biological membranes (35) may have acted synergistically with the permeabilization effect induced by PEF treatment, leading to a remarkable increase of the capacity of penetration of the solvent into the solid matrix.

#### Quantification of Vanillin and Caffeine *via* HPLC-PDA Analysis

**Figure 3:**
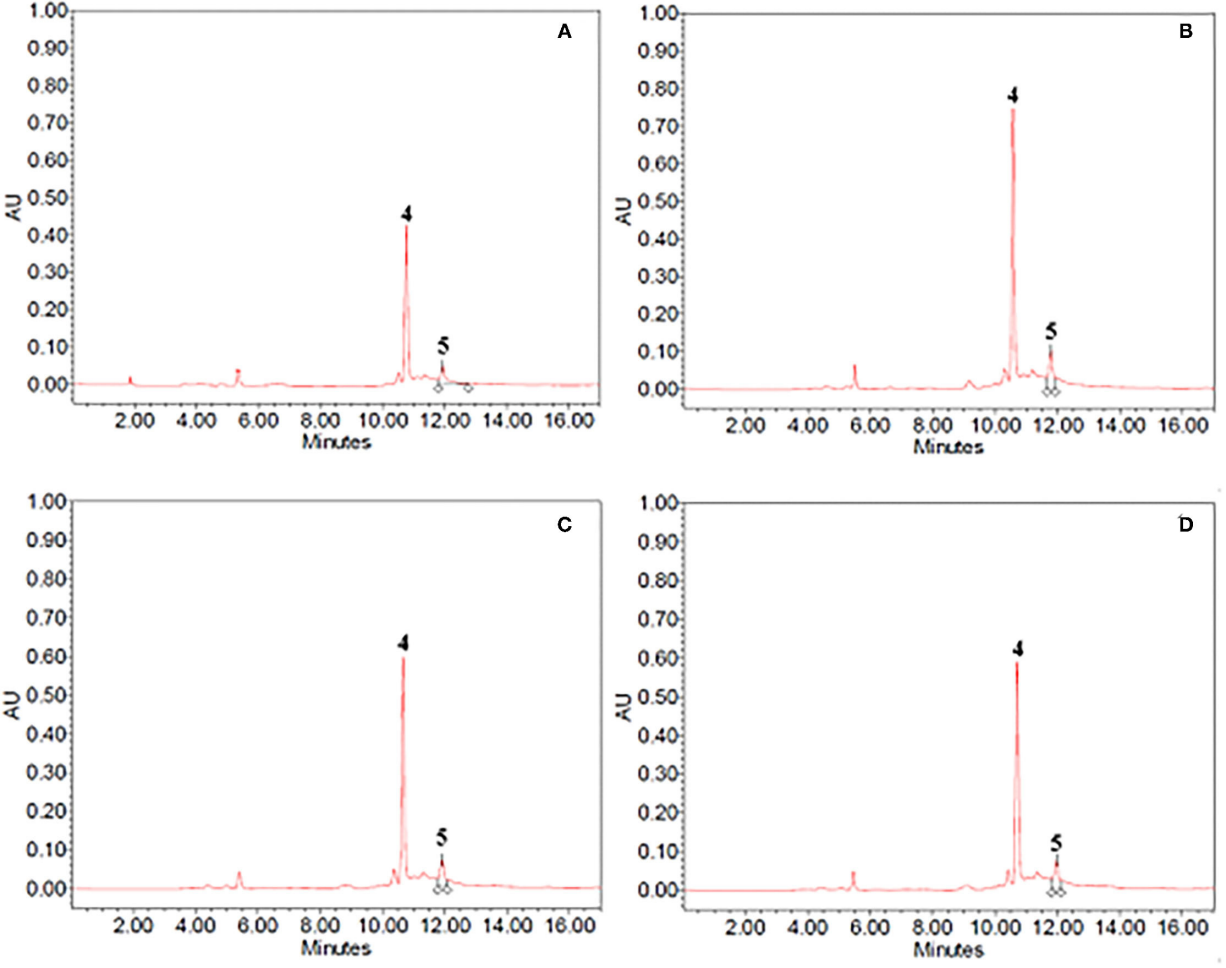
HPLC-PDA chromatograms of **(A,B)** 40% ethanol and **(C,D)** propylene glycol extracts from **(A,C)** untreated (control) and **(B,D)** PEF-treated (E_opt_ =3 kV/cm; W_T, opt_ = 20 kJ/kg) cocoa bean shells. Peak identification: theobromine (4); caffeine (5).

**Figure 4:**
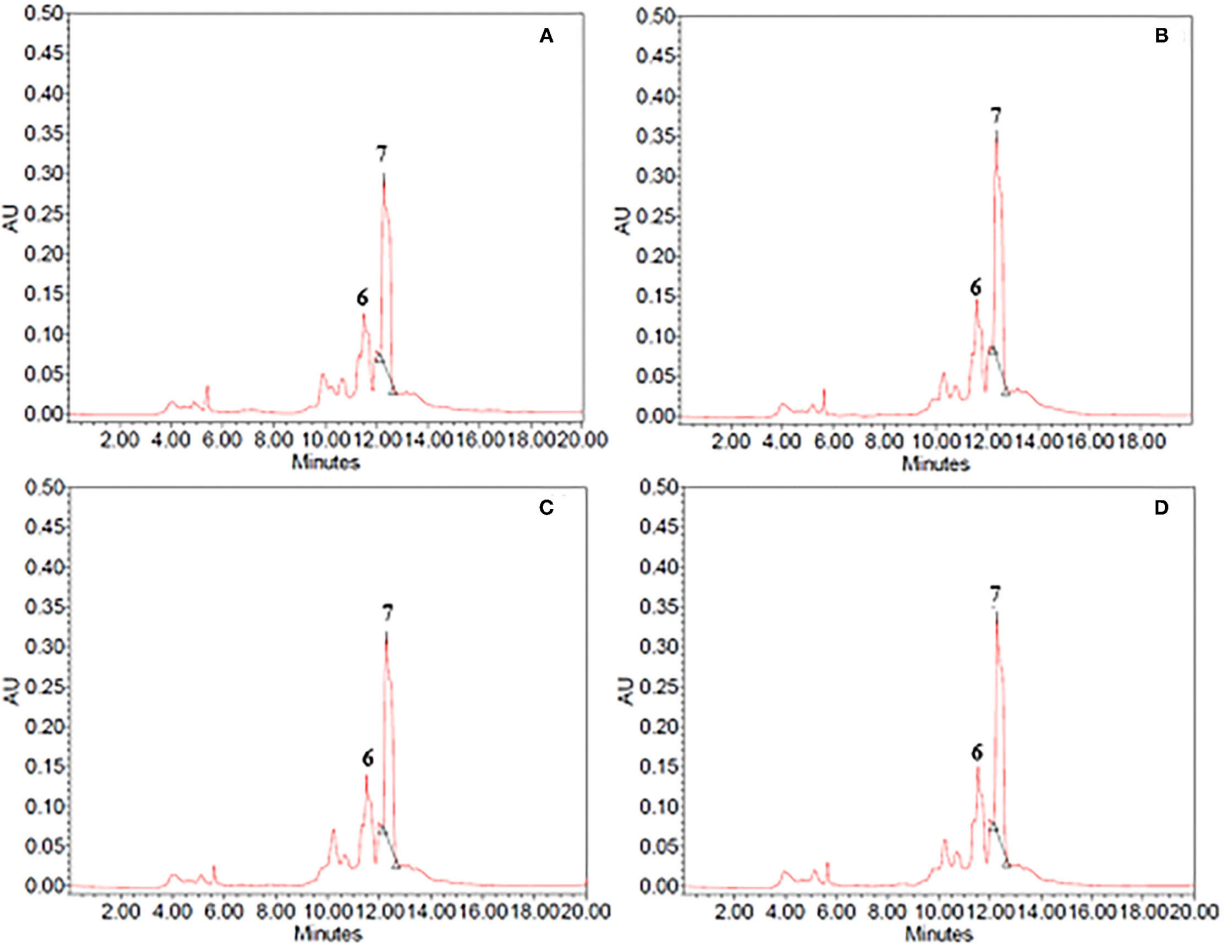
HPLC-PDA chromatograms of **(A,B)** 60% ethanol and **(B,C)** propylene glycol extracts from **(A,C)** untreated (control) and **(B,D)** PEF-treated (E_opt_ =3 kV/cm; W_T, opt_ = 20 kJ/kg) vanilla pods. Peak identification: unidentified compound (6); vanillin (7).

The composition of ethanol–water and propylene glycol extracts obtained from untreated and PEF-treated vanilla pods and cocoa bean shells, was assessed *via* HPLC-PDA analysis. The resulting chromatogram profiles are presented in Figure 3 for cocoa bean shell extracts, and Figure 4 for vanilla pod extracts, while the quantification of the bioactive compounds of interest is reported in Table 4.

**Table 4:** Concentrations (in mg/100g_DW_) of theobromine, caffeine, and vanillin detected *via* HPLC/PDA analysis in the ethanol and propylene glycol extracts from the untreated and PEF-treated cocoa bean shells and vanilla pods, respectively.

**Peak no**.	**Compound**	**Retention time (min)**	**Extraction solvent**	**Concentration (mg/100g**_**DW**_ **)**	
				**Untreated**	**PEF-treated**
4	Theobromine	10.89	Ethanol	1754 ± 89^aA^	2188 ± 105^bB^
		10.85	Propylene glycol	1875 ± 134^aB^	1903 ± 97^aA^
5	Caffeine	11.87	Ethanol	735 ± 42^aB^	987 ± 37^bB^
		11.94	Propylene glycol	197 ± 53^aA^	214 ± 41^aA^
7	Vanillin	12.30	Ethanol	621 ± 50^aA^	705 ± 12^bA^
		12.28	Propylene glycol	483 ± 11^aB^	558 ± 20^bB^

*PEF, pulsed electric field*.

*Data are expressed as means ± standard deviation*.

*PEF treatments carried out under optimal condition are reported in Table 2*.

*Different lowercase letters in the same line indicate significant differences between the samples (p ≤ 0.05). Different uppercase letters within the same column indicate significant differences between the samples (p <0.05)*.

As it can be seen, for either cocoa bean shells or vanilla pod, the chromatogram profiles of the extracts from untreated samples (Figures 3A, 4A) appeared to be similar, independent of the type of solvent.

In particular, with regard to the extraction of bioactive compounds from cocoa bean shells, the chromatogram profiles of either 40% (v/v) ethanol–water mixture (Figure 3A) or propylene glycol extracts (Figure 3C) highlighted that only two peaks, corresponding to theobromine (peak 4) and caffeine (peak 5), the two major alkaloids in cocoa, were detected. As reported in Table 4, ethanol–water mixture was most effective in extracting theobromine and caffeine, which reached, respectively, a concentration of 1,754 ± 111 mg/100g_DW_ and 735 ± 32 mg/100g_DW_ in ethanol–water mixture, and 1,975 ± 98 mg/100g_DW_ and 197 ± 13 mg/100g_DW_ in propylene glycol. It is worth noting that the concentration of theobromine and caffeine in ethanolic extracts detected in this work were consistent with those found in similar solvents by other scientists, with slight differences depending on the type of cocoa residue, and experimental protocol (10, 41–43).

As already observed for the previous matrices, regardless of the type of solvent, the application of a PEF pre-treatment to cocoa bean residues before extraction did not affect the number and type of bioactive compounds detected in the extracts. However, the permeabilization effect of the cell membrane (*Z*_*p*_= 0.82) induced by the electrical pre-treatment, remarkable (*p* < 0.05) enhanced the amount of theobromine (by 25%) and caffeine (by 34%) in ethanol–water extracts, as compared with the control extraction, while only a slight but not significant (*p* > 0.05) increase was detected when the propylene glycol was used as solvent.

In agreement with these findings, Barbosa-Pereira et al. (10) found that PEF-assisted extraction from cocoa bean shells enhanced the extractability of bioactive compounds, including theobromine and caffeine, up to ~20% (on average) when using 39% ethanol–water mixture as extracting solvent.

Concerning the vanilla extracts (Figures 4A,C), only one major peak corresponding to vanillin (peak 7) was detected at an elution time of 12.38 min in ethanol–water mixture and at an elution time of 12.30 min in propylene glycol extracts. Additionally, at least one minor and unidentified compound (peak 6) was also detected in lesser amounts in both solvents immediately before the elution time of vanillin. Furthermore, regardless of the type of solvent, the extracts obtained from untreated and PEF-pre-treated vanilla pods presented similar phenolic profiles (Figure 4), confirming once again that, under the mild PEF treatment conditions applied, there was no degradation/modification of individual phenolic compounds. This is in agreement with the observation reported by other authors for the extraction of phenolic compounds from different plant matrices (17, 28, 36, 37).

Moreover, HPLC-PDA analysis showed that the concentration of vanillin detected in ethanol–water and propylene glycol extracts of untreated samples was 621 ± 50 mg/100g_DW_ and 483 ± 11 mg/100g_DW_, respectively (Table 4). Despite the substantial amount of vanillin recovered in both investigated solvents, these results seem to confirm that 60% (v/v) ethanol–water mixture was achieved to extract vanillin from vanilla pods more effectively, as compared with propylene glycol. This could be explained by the higher polarity and penetration capacity inside the plant cell of ethanol–water mixture as compared with propylene glycol, which is consistent with findings previously reported by other scientists. For example, when Shakeel et al. (44) evaluated the solubility of vanillin in ten different environmentally green solvents, they observed a relatively high mole fraction solubility of vanillin (T = 298–318°K) in ethanol (7.94 × 10^−2^ at 298°K) and propylene glycol (7.15 × 10^−2^ at 298°K), which was significantly higher than water (1.23 × 10^−3^ at 298 K) at each temperature investigated. Similarly, in another work (45), it was observed that extraction of vanillin was higher in polar solvents such as ethanol and methanol and least in the case of non-polar solvent such as hexane, suggesting ethanol as an optimum solvent for maximum yield of vanillin.

HPLC-PDA chromatogram profiles of vanilla extracts (Figure 4) indicated that, regardless of the type of solvent, the electrical pre-treatment promoted the selective extraction of specific compounds nor caused degradation reactions. This agrees with the observations reported by other authors (13, 26, 30), who stated that PEF did not significantly alter the HPLC-PDA chromatogram profiles of different plant extracts, due to the relatively mild intensity of the applied treatment.

However, it is worth noting that, in comparison with the control sample and regardless of the type of solvent, the permeabilization effect of the cell membrane (*Z*_*p*_= 0.82) induced by PEF pre-treatment increased the peak area of vanillin (peak 7), whereas no appreciable changes could be detected in the peak area of the unidentified compound (peak 6). In particular, coherently with the results of Figures 1, 2, the application of PEF pre-treatment caused a remarkable increment of the concentration of vanillin by 14 and 16% in ethanol–water mixture and propylene glycol extracts, respectively, as compared with control extraction.

## Conclusions

The results of this work demonstrated that the application of PEF pre-treatment of moderate intensity (3–5 kV/cm) and relatively low energy input (15–40 kJ/kg) before solid liquid extraction (SLE) with green solvents, such as ethanol–water mixture and propylene glycol, can represent an environmentally friendly approach to intensify the extractability of valuable intracellular compounds, such as linalool from vermouth mixture, limonene from orange peels, vanillin from vanilla pods, and theobromine and caffeine from cocoa bean shells.

The higher recovery yields of all the compounds of interest detected in ethanol–water mixture extracts in comparison with propylene glycol extracts, indicates a better capability of this solvent to penetrate the cells of the processed plant tissue and to solubilize a greater amount of target intracellular compounds of a wide range of polarity.

Pulsed electric field pre-treatment at the optimized process conditions achieved for each plant matrix, was able to induce a sufficiently high level of tissue permeabilization, but to an extent depending on the type of matrices, which exhibited the following trend (in decreasing *Z*_*p*_ order): vanilla pods, cocoa bean shells > vermouth mixture > orange peels. Interestingly, for all investigated matrices, the cell disintegration induced by PEF pre-treatment further enhanced the extraction yield of the target compounds, without affecting the number and type of bioactive compounds detected in the extracts.

Finally, the combination of PEF with an ethanol-based solvent that can affect the phospholipid bilayer of biological membranes can represent a valuable green and effective approach, especially for those plant matrices, such as orange peels, whose cell envelope showed higher resistance to permeabilization effects of PEF.

## Data Availability Statement

The raw data supporting the conclusions of this article will be made available by the authors, without undue reservation.

## Author Contributions

GP, AR, and GF contributed to the conception and design of the study. SC was in charge of performing chemical and statistical analysis and wrote the first draft of the manuscript. GP and SC performed the experiments. GP and GF supervised the study. All authors contributed to manuscript revision, read, and approved the submitted version.

## Conflict of Interest

The authors declare that the research was conducted in the absence of any commercial or financial relationships that could be construed as a potential conflict of interest.

## Publisher's Note

All claims expressed in this article are solely those of the authors and do not necessarily represent those of their affiliated organizations, or those of the publisher, the editors and the reviewers. Any product that may be evaluated in this article, or claim that may be made by its manufacturer, is not guaranteed or endorsed by the publisher.
