# Supplementary material for: Pulsed Electric Field-Assisted Extraction of Aroma and Bioactive Compounds From Aromatic Plants and Food By-Products
Source: Front Nutr. 2022 Jan 27;8:792203. doi: 10.3389/fnut.2021.792203 (PMC8829011; doi:10.3389/fnut.2021.792203)
Supplement: Supplementary file 1 [file Image_1.pdf]

SUPPLEMENTARY MATERIAL

Pulsed electric field-assisted extraction of aroma and bioactive compounds from aromatic plants and food by-products

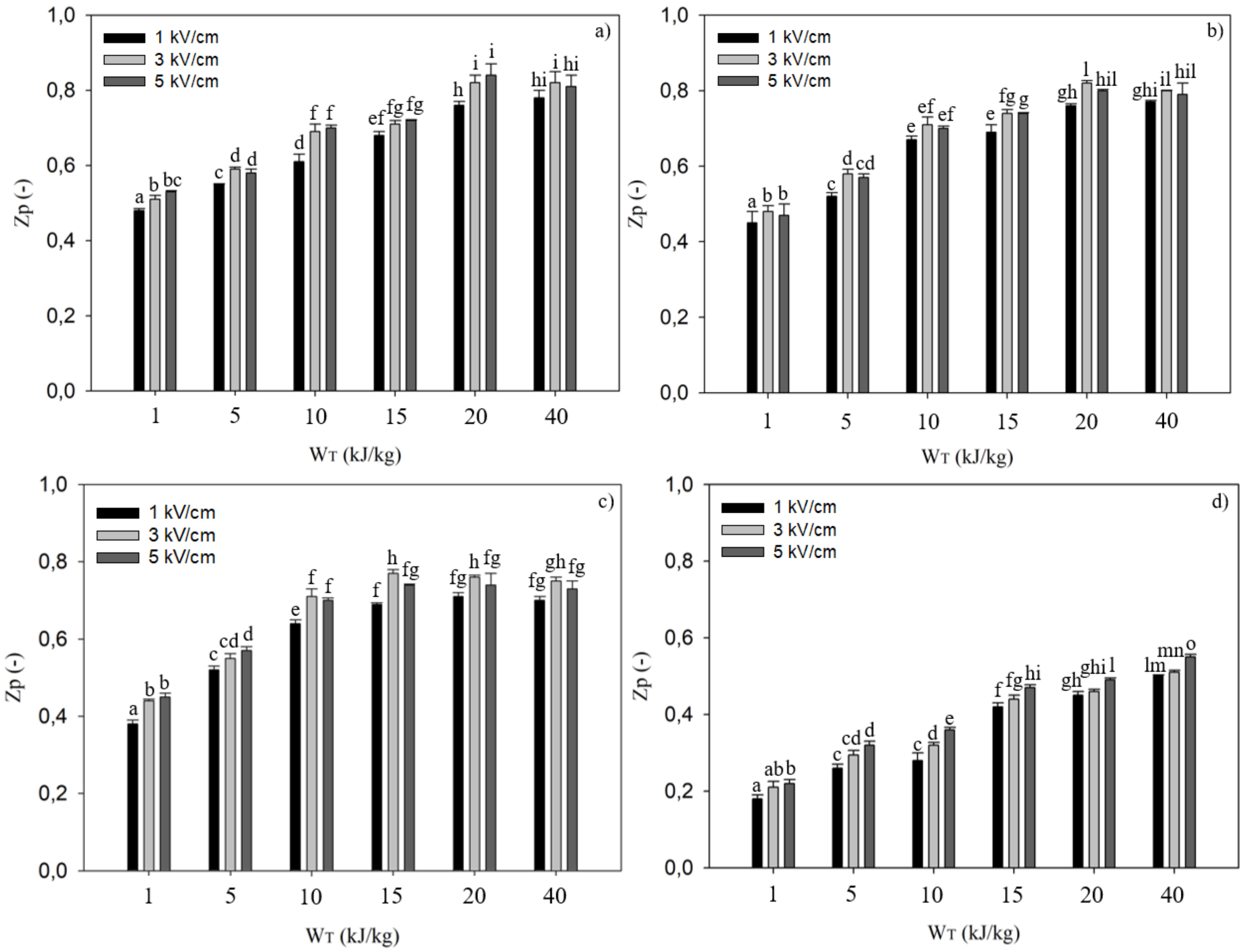

Figure S1 Cell disintegration index (Z_P) of vanilla pods (a), cocoa bean shells (b), vermouth mixture (c), and orange peels (d) as a function of electric field strength E (1, 3, 5 kV/cm) and total specific energy input W_T (1, 5, 10, 15, 20, 40 kJ/kg). Different letters above the bars indicate significant differences between the mean values ($p \leq 0.05$).
